# The right to rehabilitation for people with dementia: A co‐design approach to barriers, solutions and strategies

**DOI:** 10.1002/alz.087169

**Published:** 2025-01-09

**Authors:** Natasha Layton, Catherine Devanny, Keith Hill, Kate Swaffer, Grant Russell, Lee‐Fay Low, Angel Lee, Monica Cations, Helen Skouteris, Claire O'Connor, Taya Collyer, Barbara Barbosa Neves, Nadine Andrew, Terry Haines, Velandai K Srikanth, Alan Petersen, Michele L Callisaya

**Affiliations:** ^1^ Monash University, Melbourne, VIC Australia; ^2^ University of South Australia, Adelaide, SA Australia; ^3^ The University of Sydney, Sydney, NSW Australia; ^4^ Flinders University, Adelaide, SA Australia; ^5^ University of New South Wales, Sydney, NSW Australia; ^6^ HammondCare, Sydney, NSW Australia; ^7^ Peninsula Clinical School, Central Clinical School, Monash University, Melbourne, VIC Australia; ^8^ University of Sydney, Sydney, NSW Australia; ^9^ Frankston Hospital, Peninsula Health, Melbourne, VIC Australia; ^10^ Menzies Institute for Medical Research, University of Tasmania, Hobart, TAS Australia; ^11^ Peninsula Health, Melbourne, VIC Australia; ^12^ National Centre for Healthy Ageing, Melbourne, VIC Australia

## Abstract

**Background:**

People with dementia of all ages have a human right to equal access to quality health care. Despite evidence regarding its effectiveness, many people living with dementia lack access to evidence‐based rehabilitation for promoting function and quality of life. The aims of this study were to 1) explore barriers to access to dementia rehabilitation; and 2) identify solutions which improve access to rehabilitation.

**Method:**

People living with dementia and care partners (n = 13) and health professionals (n = 13) were invited to participate. Experience based co‐design across three virtual workshops was used to understand barriers and solutions to improve access to rehabilitation treatments. Socio‐ecological analyses, using the Levesque Access to Health Care Framework, were applied to findings regarding barriers and to assist selection of solutions.

**Result:**

There was high attendance (92.3%) across the three workshops. Barriers were identified at a person‐level (including lack of knowledge, difficulty navigating the health, aged‐care and disability system, transport, and cost) and health service‐level (including health professional low knowledge and negative attitudes, inequitable funding models and non‐existent or fragmented services). Solutions focused on widespread education and training in the area of dementia rehabilitation, including ensuring people with dementia and their care partners know about rehabilitation therapies and that health professionals, aged care and disability co‐ordinators know how to refer and deliver interventions. Dementia care navigators, changes to Commonwealth aged care and Medicare funding models, and specific dementia rehabilitation programs were also recommended.

**Conclusion:**

Barriers to accessing rehabilitation for people with dementia exist at multiple levels and will require a whole community and systems approach to ensure change.

